# Supplementary material for: Conformational Plasticity of Hepatitis B Core Protein Spikes Promotes Peptide Binding Independent of the Secretion Phenotype
Source: Microorganisms. 2021 Apr 29;9(5):956. doi: 10.3390/microorganisms9050956 (PMC8145704; doi:10.3390/microorganisms9050956)
Supplement: Supplementary file 1 [file microorganisms-09-00956-s001.zip › microorganisms-1190863-supplementary.pdf]

Supplemental Information

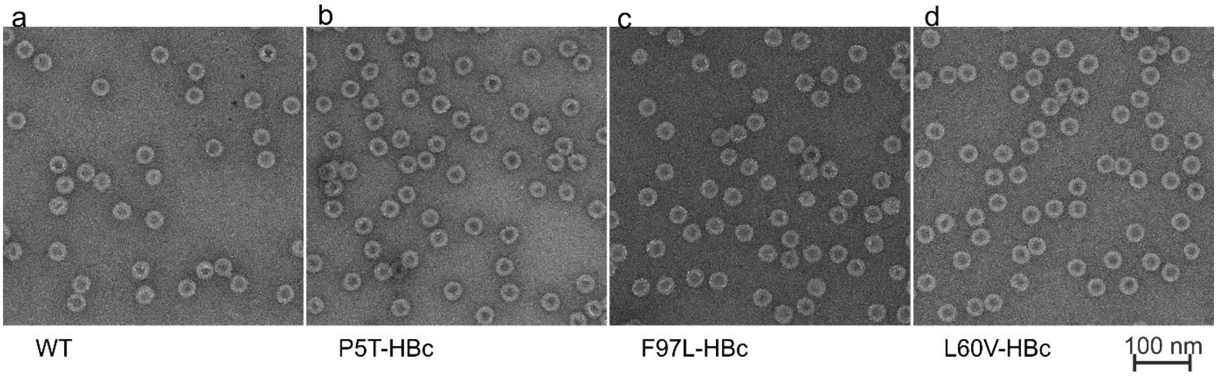

Figure S1 Electron micrographs of purified, negatively stained HBc-capsids. (a) WT-HBc, (b) P5T-HBc, (c) F97L-HBc and (d) L60V-HBc. The samples are the same as shown in SDS-PAGE and NAGE in Figure 1.

Table S1 Instrumental conditions of ITC experiments

Number of injections	20	Parameters of following 19 injections	
Cell Temperature (°C)	20 (37 for P1)	Volume	2 µl
Reference power (µCal/s)	11	duration (s)	4
Initial delay (s)	60	Spacing (s)	180
Stirring speed	600	Filter period (s)	5
Feedback mode	high	Cell volume (ml)	0,2009
First injection	0,5 µl for 1 s	180 s spacing	Filter 5

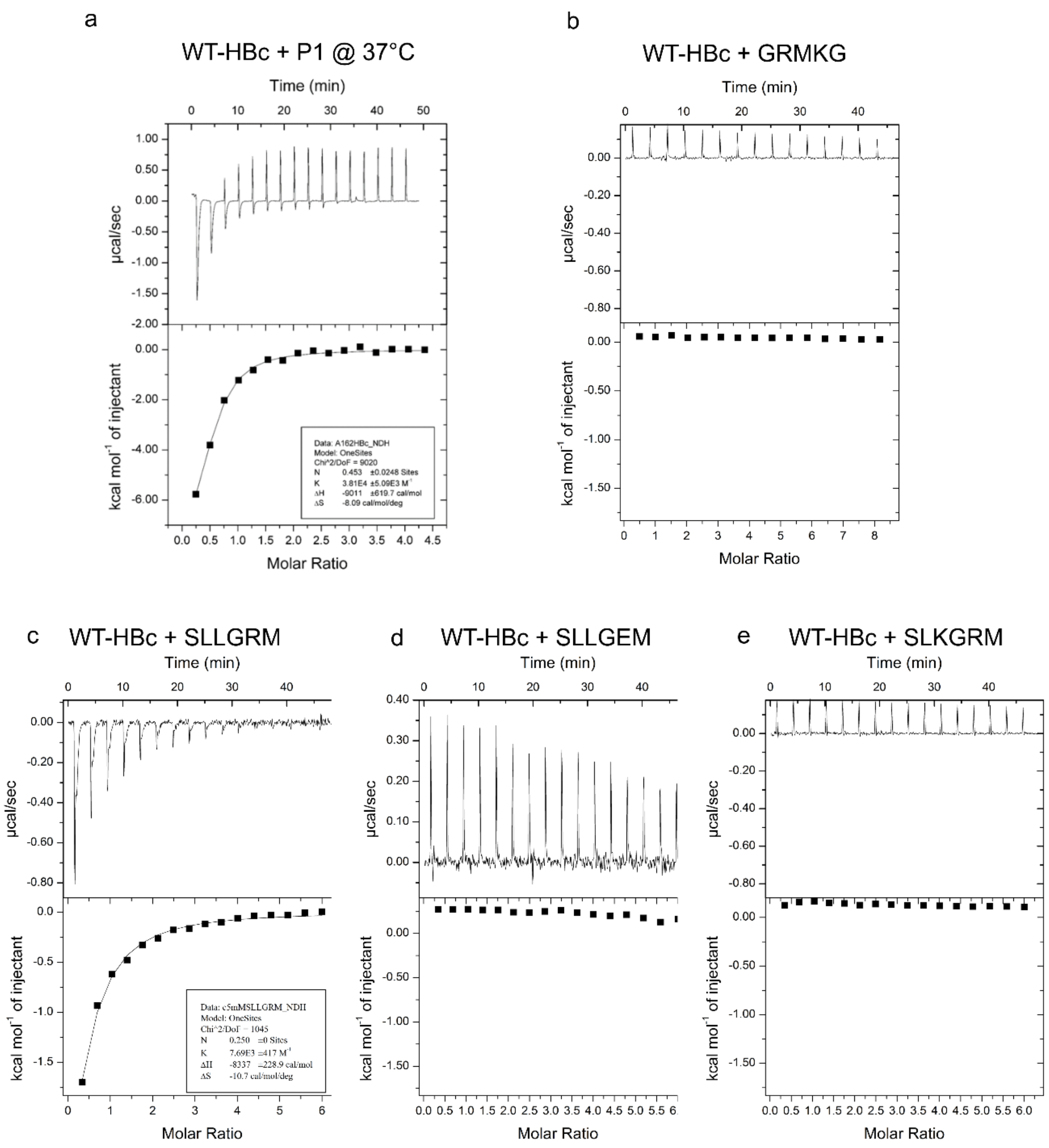

Figure S2 Isothermal Titration Calorimetry of WT-HBc with different peptides. The peptides were: (a) P1 (‘MHRSLLGRMKGA’), (b) ‘GRMKG’, (c) ‘SLLGRM’, (d) ‘SLLGEM’ and (e) SLKGRM. These experiments were done as single repeat experiments (table 1). The titration in (a) was done in ITC-buffer 1 at 37°C. All other experiments were done at 20°C in ITC-buffer 2. Binding was only observed for P1 and ‘SLLGRM’. P1 titration showed a significant endothermic contribution at higher P1 concentrations, which we attribute to aggregation of P1 and capsids.

Table S2: ***Summary of imaging conditions for cryo EM data acquisition.*** All movies were acquired at 300 kV with the same exposure rate 16 e/(Å²s) but with different total exposure. A characteristic micrograph for each sample after motion correction is shown in the appendix.
(P2="GSLLGRMKGA"; P1="MHRSLLGRMKGA"; 1): peptides were added at 10-fold molar excess; 2): capsids were taken from the endpoint of titration in the ITC-experiment.

Sample	Movies per hole	targeted defocus /µm	Exposure time /s	exposure /(e/Å²)	Frames per movie
L60V	3	0.8-1.4	2.0	32	20
L60V+P2 1)	3	0.8-1.5	5.0	82	40
P5T	3	0.8-1.4	2.5	40	20
P5T+P1 1)	2	0.8-1.5	5.2	83	40
P5T+P2 1)	2	0.7-1.2	3.1	49	25
WT	3	0.6-1.6	2.5	40	40
WT+P1 1)	2	0.8-1.8	5.2	83	40
WT+P2 1)	2	0.8-1.4	3.3	52	25
WT+P2 2)	3	0.6-1.6	2.6	40	40
WT+SLLGRM 2)	3	0.6-1.6	2.4	40	40
WT+GRMKG 1)	3	0.6-1.6	2.4	40	40
F97L+P1 1)	2	0.8-1.5	5.2	83	40
F97L+P2 1)	3	0.8-1.5	5.0	80	40

Table S3 Summary of Image Processing; (P2="GSLLGRMKGA"; P1="MHRSLLGRMKGA". [1)]: peptides were added at 10-fold molar excess; [2)]: capsids were taken from the endpoint of titration in the ITC-experiment.

For each sample, the most populated class averages, a Fourier-shell-correlation plot and an equatorial slice of the final map are shown in the appendix.

Sample	Movies in data set	No of selected particles	No of particles in final map	Resolution /Å²	B-factor map /Å²
L60V	3618	165231	119234	3.2	158
L60V+P2[1)]	2694	244935	150699	3.0	153
P5T	2421	169111	61896	3.2	139
P5T+P1[1)]	2453	144210	75833	2.9	120
P5T+P2[1)]	1986	126953	74555	3.2	149
WT	2423	228237	142173	2.8	106
WT+P1[1)]	1230	43184	10103	4.0	165
WT+P2[1)]	1578	110918	66884	3.0	141
WT+P2[2)]	2745	251669	126554	2.8	108
WT+GRMKG	2358	226729	133100	2.5	91
WT+SLLGRM[2)]	2547	273232	157183	2.8	119
F97L+P1[1)]	1110	53409	29437	2.9	95
F97L+P2[1)]	1146	105062	73779	3.1	138

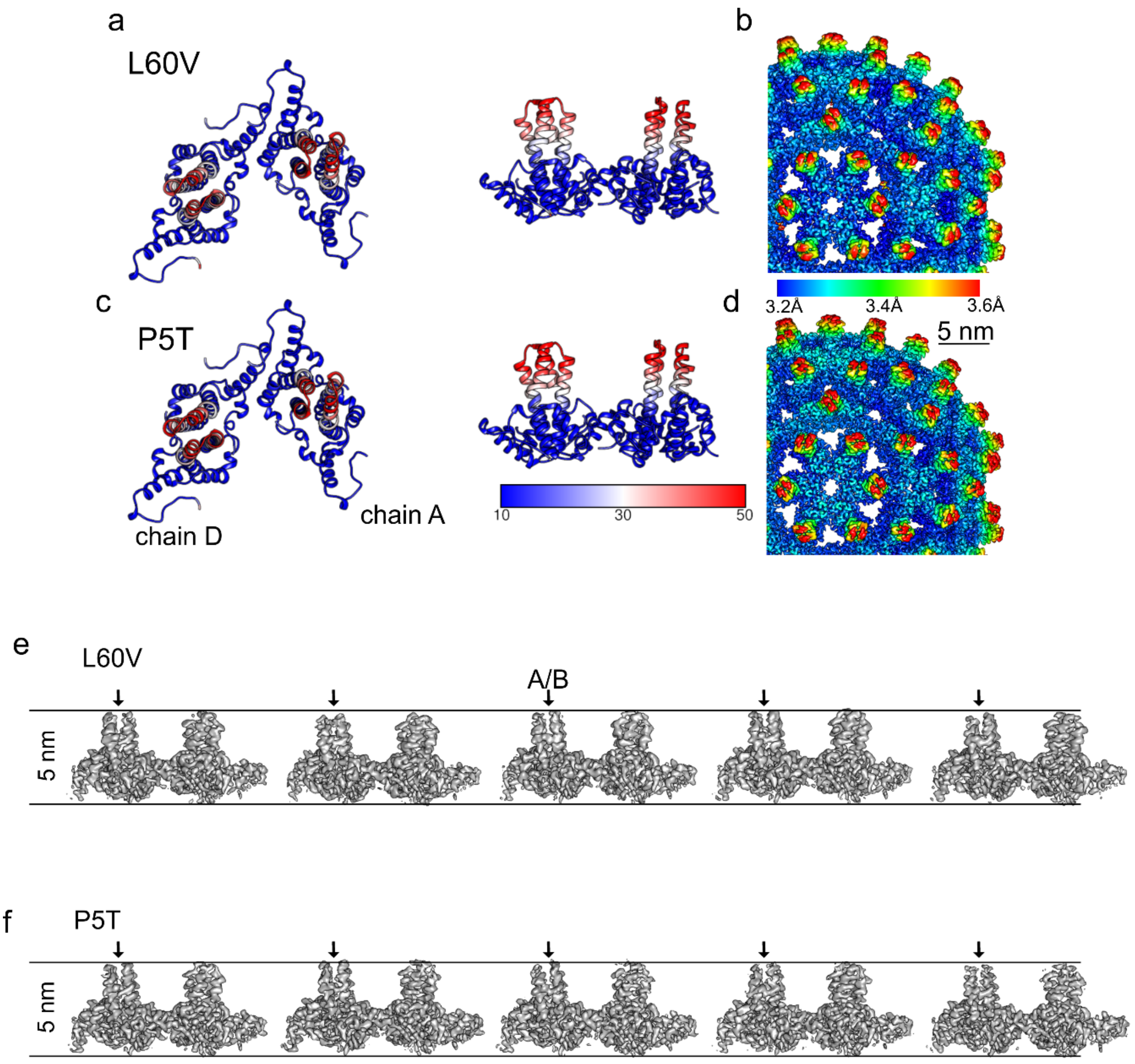

Figure S3 Local Variations of the asymmetric unit in capsids of L60V-HBc (a,b,e) and P5T-HBc (c,d,f). (a) and (c) show the models of the asymmetric unit of L60V-HBc and P5T-HBc coloured with their per residue B-factors determined by Phenix real-space refinement against density scaled maps (same radial intensity profile of power-spectra). The colour-key for the B-factors/Å² is given below. The views in the left and right panel are related by a 90° rotation around the horizontal axis. (c) and (d) show the outer capsid surface coloured by the local resolution as indicated by the colour key. (e) and (f) show the class averages derived by classification of the symmetry related asymmetric units of the capsids without alignment. The two lines indicate positions with the same radial spacing. Their distance is 5 nm. The classes show movements of 1-2 Å relative to their radial spacing. The arrow identifies the same symmetry related coordinate on the capsid-surface for the different classes. The class averages are scaled to the same radial intensity profile of their power spectra.

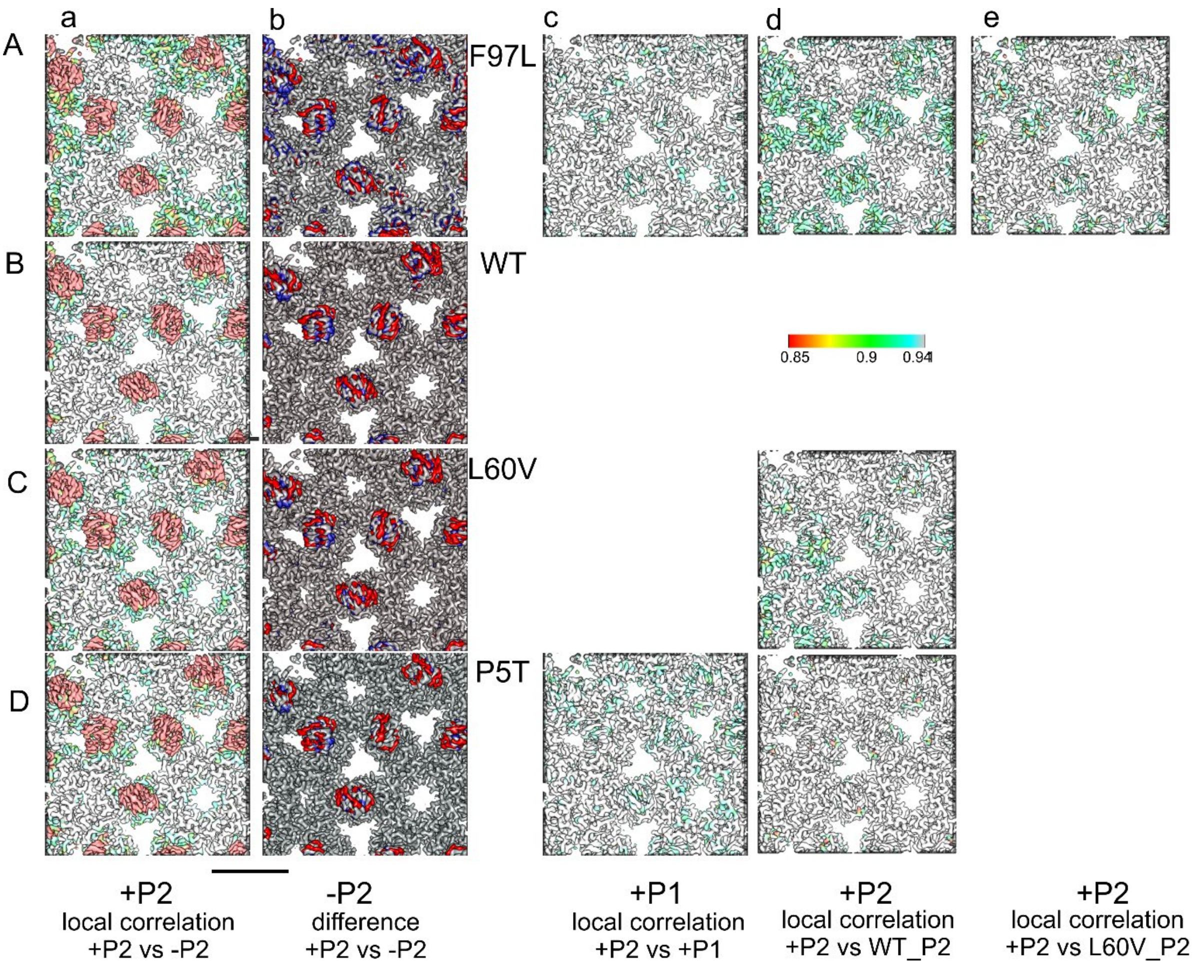

Figure S4 Comparison of density scaled maps of HBc-variants with and without bound peptides. The different rows show close-ups of the outer surface of the capsids at a local 3-fold symmetry axis for F97L-HBc (A), WT-HBc (B). L60V-HBc (C) and P5T-HBc (D). The variants are shown with bound P2 in columns (a), (d) and (e) and with bound P1 in column (c) and without bound peptide in column (b). The surface is coloured according to the local correlation between maps of the same HBc-variant with and without P2 in row (a), of the same HBc-variant with P1 or P2 in column (b), of HBc-variant and WT-HBc both bound with P2 in row (d). Row (e) is coloured with the local correlation between L60V-HBc+P2 and F97L-HBc+P2. The colour key is shown in panel Bd and is the same as in Figure 3 and Figure S5 and S6. In row (b) the difference between the HBc-variant with and without bound peptide is shown in colour as surface representation at a threshold of 2/3 of that used for the HBc-variant. Positive differences are shown in red and negative densities in blue. Small, discontinuous speckles with a size of less than 10 Å³ are removed (Chimera option hide dust). Peptide binding is evidenced by the low local correlation between maps with and without bound peptides and by the differences at the tips of the spikes. Please note (Ad) and (Ba) are also shown in Figure 4.

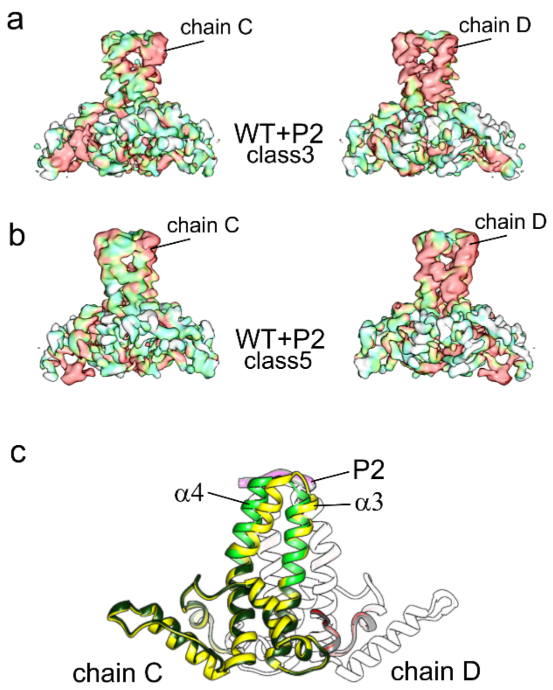

Figure S5 chain C and D of the most diverging classes of WT-HBc + P2. (a) and (b) show surface representations of the most diverging class-averages of class3 (a) and class (5) of the CD-dimer after classification of the asymmetric unit without further alignment. The surface is coloured with the local correlation between the density scaled maps of the class averages. The same colour key applies as in Figure S4. The two views in the left and right panel are related by a 180° rotation around the vertical axis. (c) shows the model of the CD-dimer for the two classes. Chain C is shown in yellow for class3 and in green for class5.

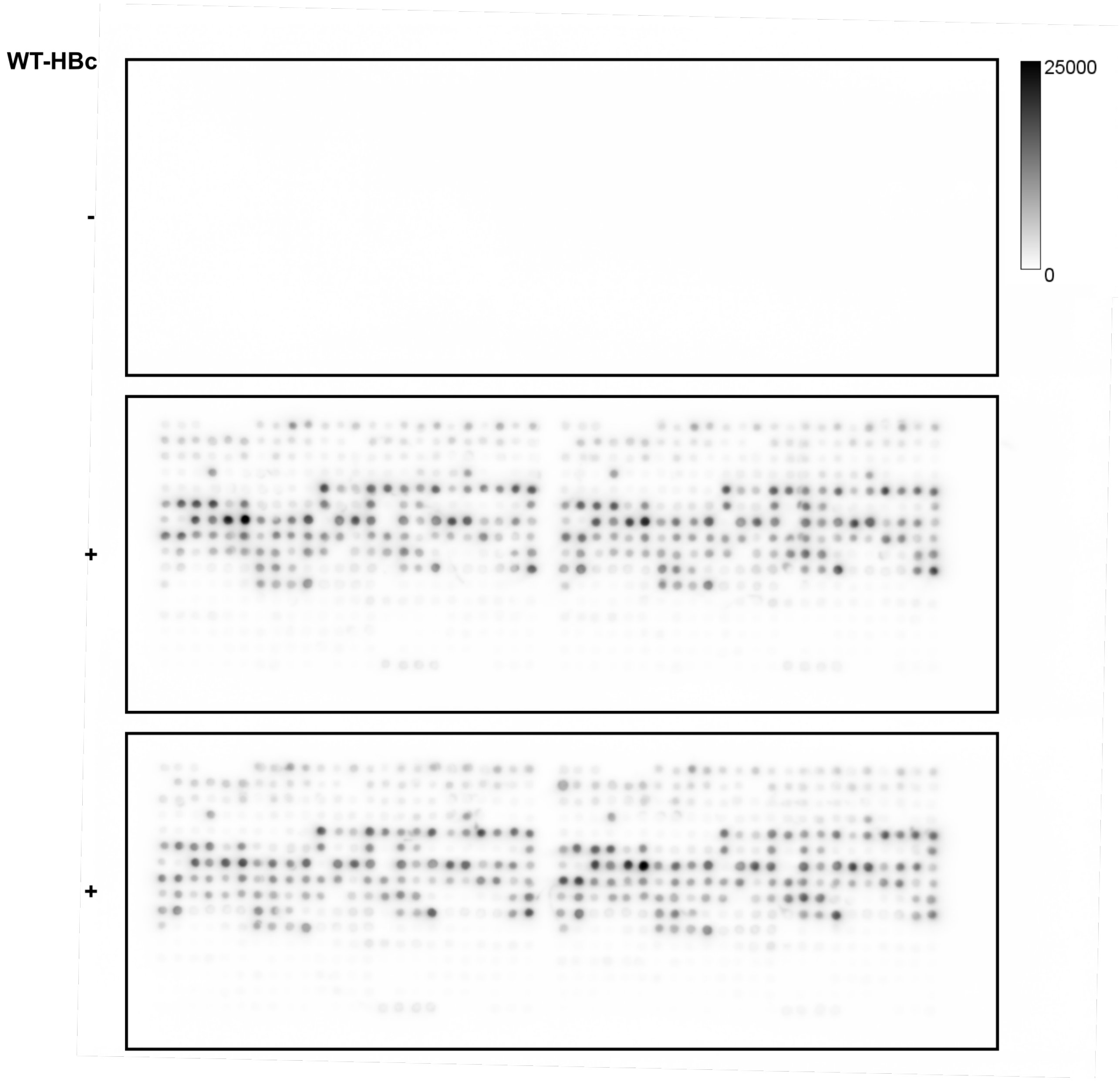

Figure S6 Peptide microarray of P2-permutations and terminal truncations probed with WT-HBc. Calibration bar represents the raw greyscale values. Lower slides were incubated with 55 nM (monomer equivalent) of WT-HBc. All slides were incubated with a primary 1:2500 diluted mAb16988 (Anti-HBc, MilliporeSigma) and a secondary 1:5000 diluted HRP-coupled Anti-mouse antibody (31430, Invitrogen). Chemiluminescent readout is visible only on the lower slides that were incubated with WT-HBc.

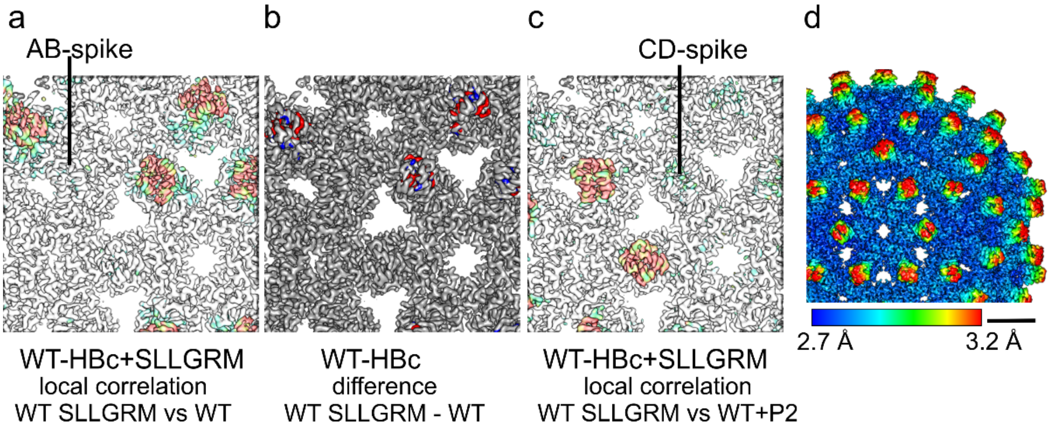

Figure S7 WT-HBc with bound SLLGRM. (a) and (c) show WT-HBc with bound SLLGRM. The maps are coloured according to the local correlation with WT-HBc in (a) and with WT-HBc+P2 in (c). The colour key for the local correlation is the same as in Figure S4. Note that in (a) the tips of the CD-spikes with the bound SLLGRM have lower local correlation than the AB-spikes (one of the AB-spikes is indicated). In (c) it is vice versa (one of the CD-spikes is indicated). (b) shows the surface of WT-HBc in grey with the differences between WT-HBc+SLLGRM and WT-HBc superposed. Positive differences are shown in red, negative differences are shown in blue. Note, differences are mainly present at the tips of the CD-spikes but not at the tips of the AB-spikes. (d) shows a view of the outer surface of the WT-HBc+SLLGRM capsid. The map was filtered according to its local resolution and coloured with the local-resolution as according to the colour-key. The scale bar indicates 5 nm. Please note, panel (a) is also shown in Figure 7.

Supplemental Information 1

Purity of the in-house made, amidated peptides (SLLGRM, SLLKEM, SLKGRM) used for ITC

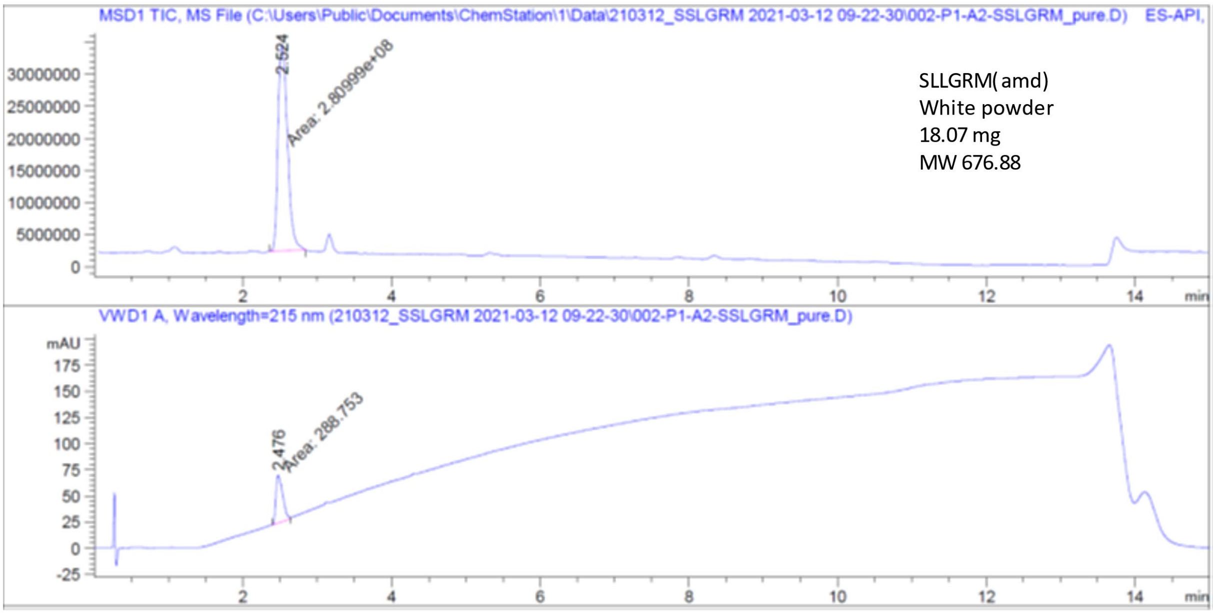

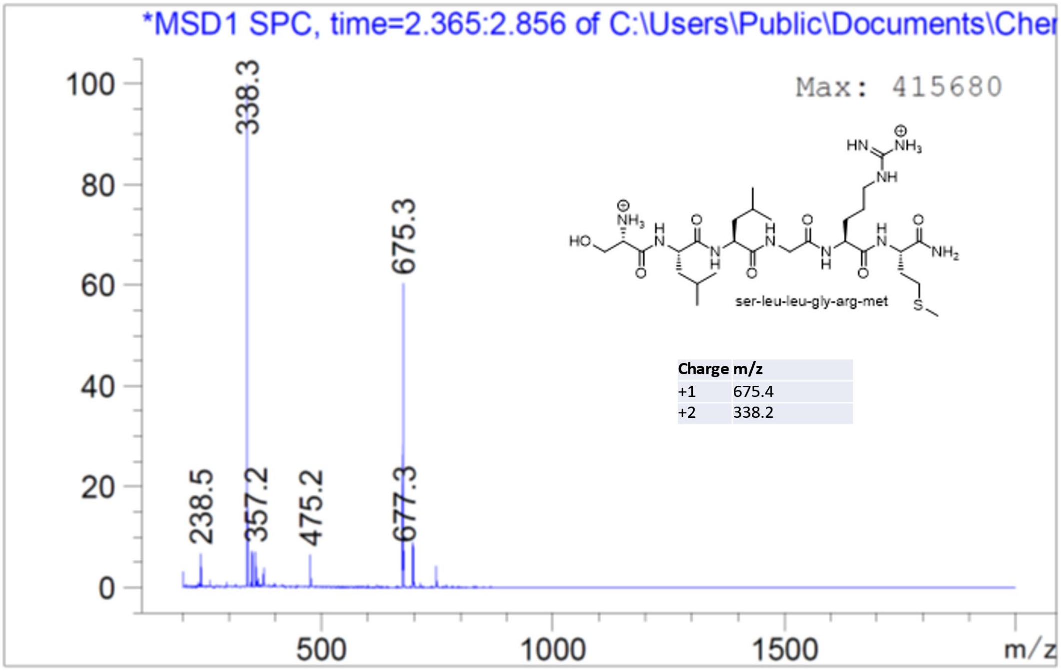

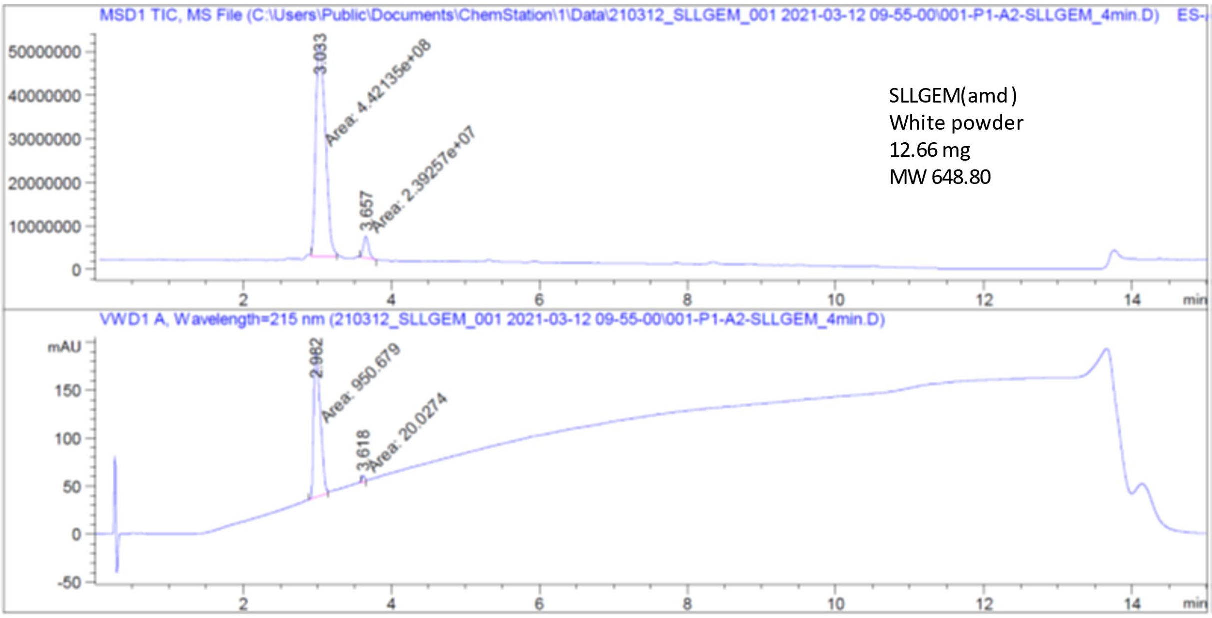

Peak 1
Area 950U
97.9%

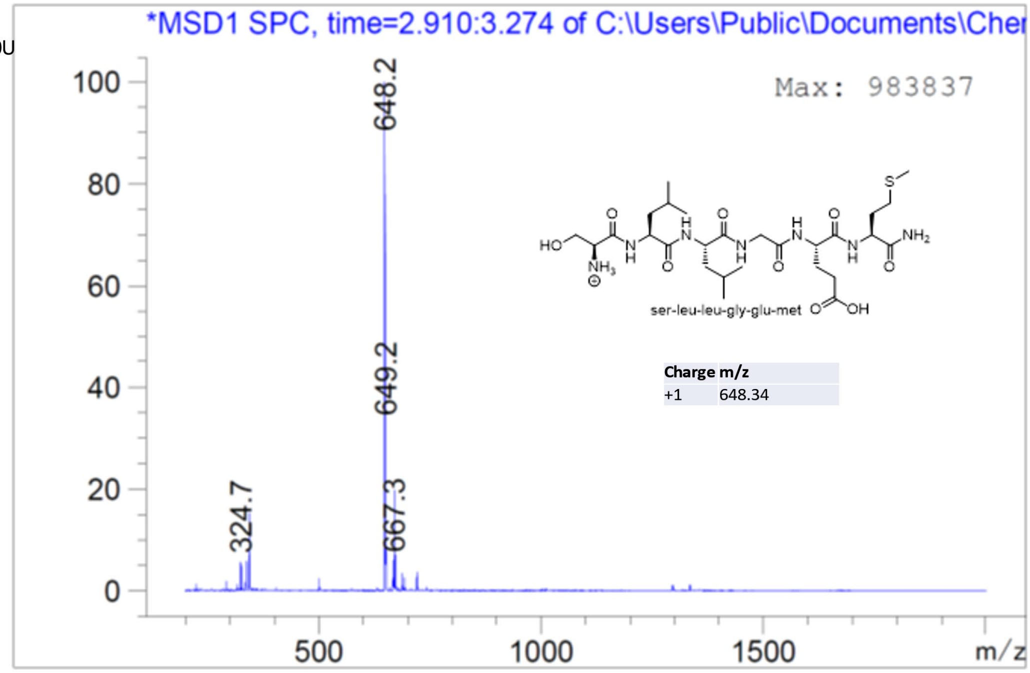

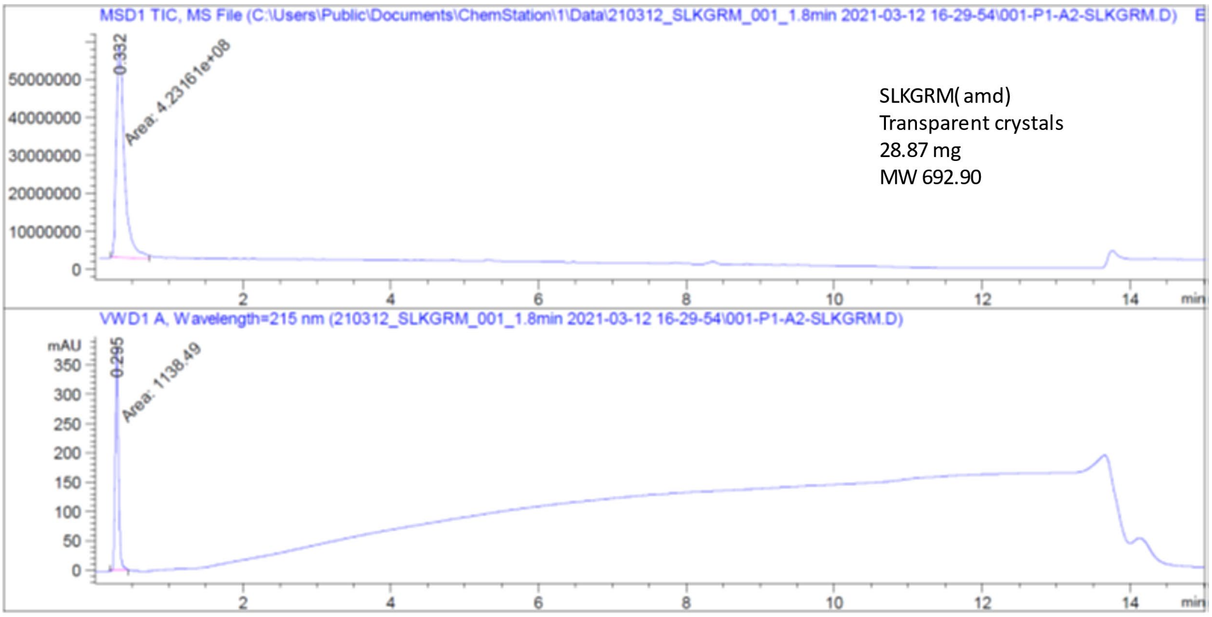

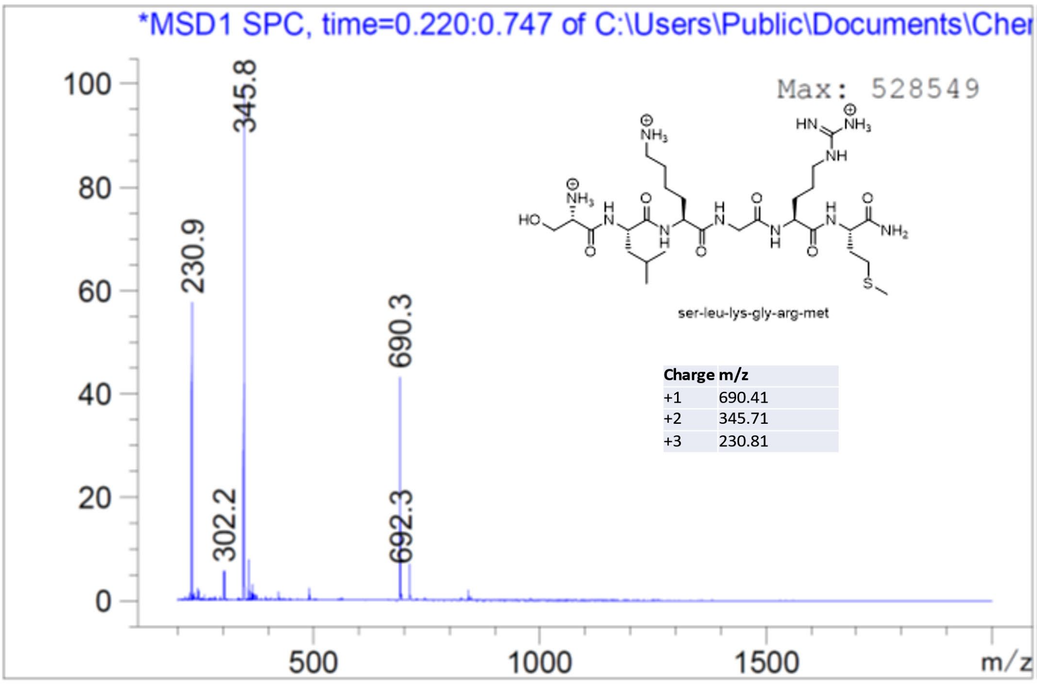

Charge	m/z
+1	690.41
+2	345.71
+3	230.81

Supplemental Information 2

Micrographs, class averages, map quality and Fourier shell correlation

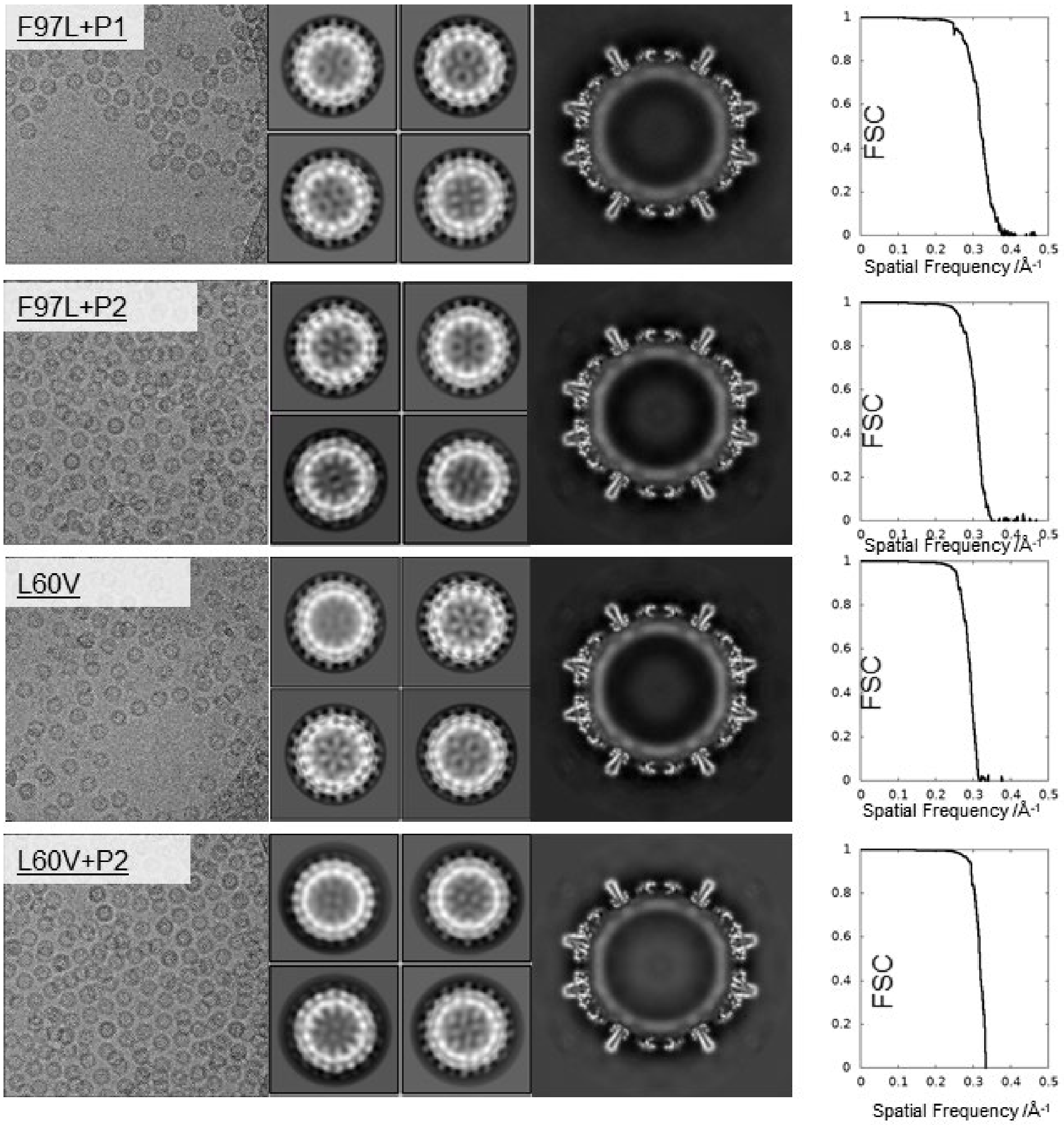

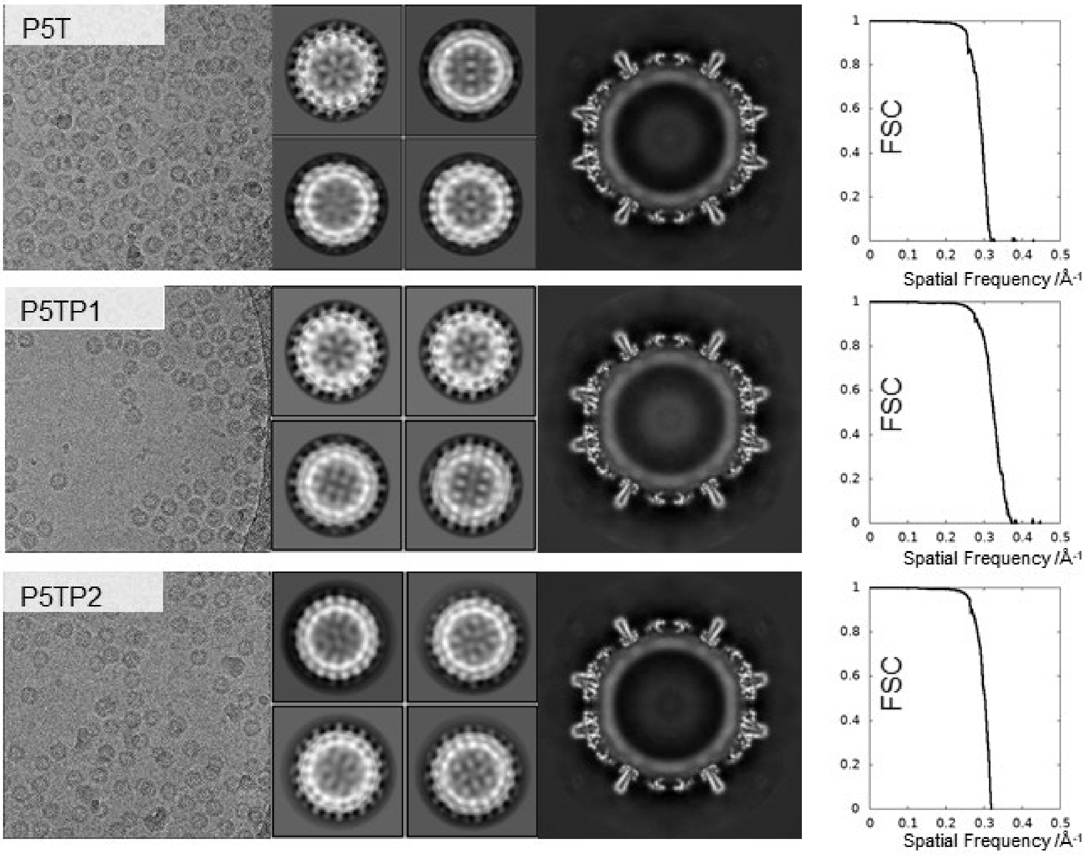
P5T
P5TP1
P5TP2
FSC
Spatial Frequency /Å⁻¹
0
0.2
0.4
0.6
0.8
1
0.1
0.3
0.5

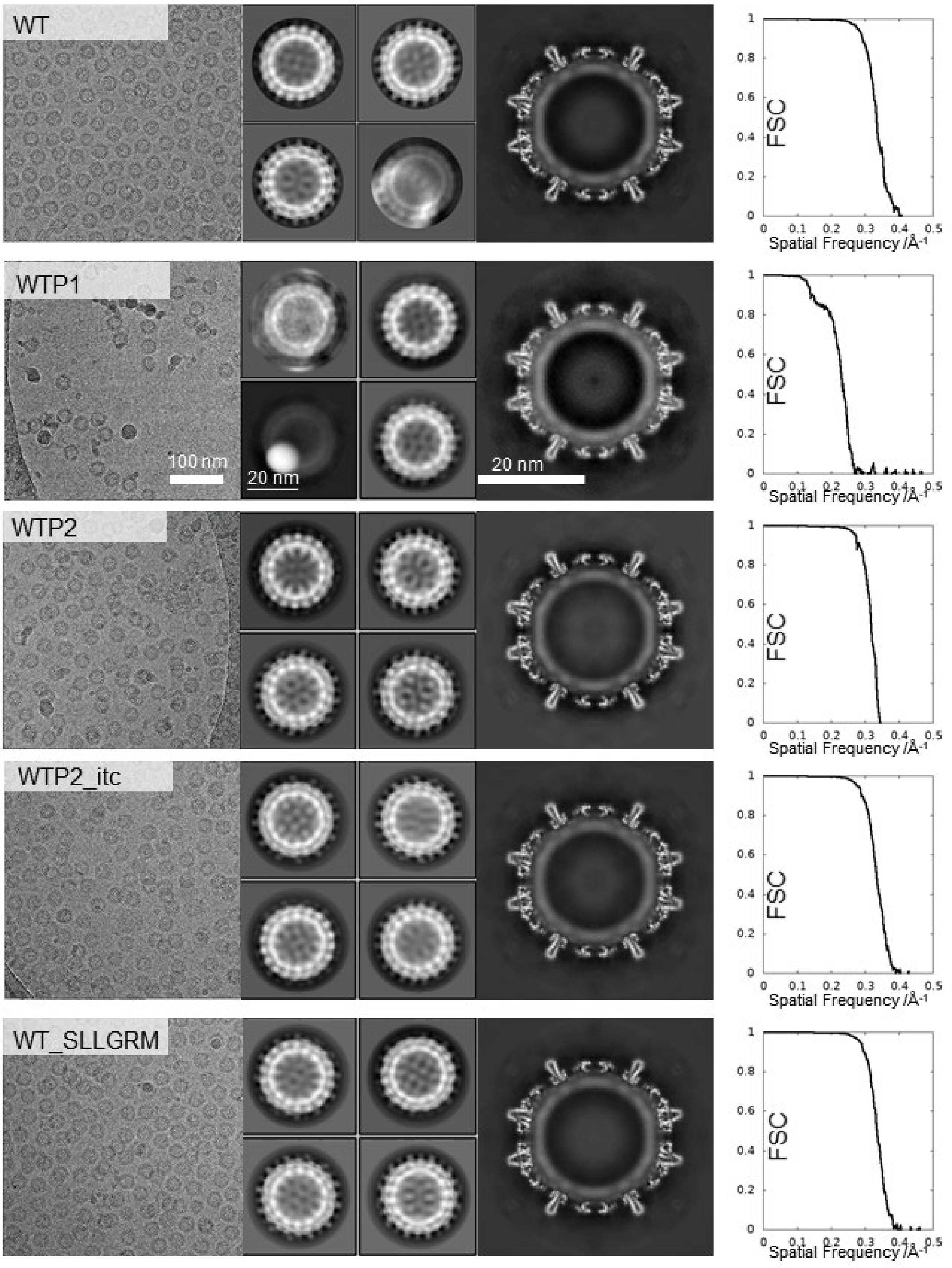

For each data set a characteristic micrograph after motion correction, the four most populated classes after auto-picking, an equatorial slice of the final EM-map before B-factor sharpening and the Fourier-shell correlation plots are shown. The Fourier-shell-correlation is corrected for the effects of the mask.
